# Evaluating the effectiveness and implementation of evidence-based early-life nutrition interventions in a community setting a hybrid type 1 non-randomized trial – the Nutrition Now project protocol

**DOI:** 10.3389/fendo.2022.1071489

**Published:** 2023-01-10

**Authors:** Nina Cecilie Øverby, Elisabet Rudjord Hillesund, Sissel Heidi Helland, Christine Helle, Andrew Keith Wills, Admassu Nadew Lamu, Natalie Garzon Osorio, Henrik Lian, Torunn Iveland Ersfjord, Wim Van Daele, Tormod Bjørkkjær, Erlend Nuland Valen, Mekdes Kebede Gebremariam, Erik Grasaas, Charlotte Kiland, Ulrica von Thiele Schwarz, Marianne Hope Abel, Penny Love, Karen Campbell, Harry Rutter, Mary Elizabeth Barker, Frøydis Nordgård Vik, Anine Christine Medin

**Affiliations:** ^1^ Department of Nutrition and Public Health, Faculty of Health and Sport Sciences, Priority Research Centre Lifecourse Nutrition, University of Agder, Kristiansand, Norway; ^2^ Department of Community Medicine and Global Health , Institute of Health and Society, University of Oslo, Oslo, Norway; ^3^ Department of Political Science and Management, Faculty of Social Sciences, University of Agder, Kristiansand, Norway; ^4^ School of Health, Care and Social Welfare, Mälardalen University, Västerås, Sweden; ^5^ Procome, Medical Management Centre, LIME, Karolinska Institutet, Stockholm, Sweden; ^6^ Centre for Evaluation of Public Health Measures, Norwegian Institute of Public Health, Oslo, Norway; ^7^ Institute for Physical Activity and Nutrition, School of Exercise and Nutrition Science, Deakin University, Geelong, VIC, Australia; ^8^ Department of Social and Policy Sciences, University of Bath, Bath, United Kingdom; ^9^ School of Health Sciences, University of Southampton, Southampton, United Kingdom; ^10^ MRC Lifecourse Epidemiology Centre, University of Southampton, Southampton General Hospital, Southampton, United Kingdom

**Keywords:** digital diet intervention, early life obesity prevention, maternal and child health care, implementation, feeding practices, municipality scale up, the Nutrition Now project protocol

## Abstract

Disappointingly few efficacious health interventions are successfully scaled up and implemented in real world settings. This represents an evidence-to-practice gap, with loss of opportunity to improve practice. Aiming to improve nutrition in the first 1000 days of life, we have combined four efficacious dietary interventions into a single adapted digital resource (Nutrition Now) for implementation in a Norwegian community setting. Nutrition Now targets pregnant women and parents of 0–2-year-olds with messages focusing on healthy dietary behaviours. Early childhood education and care (ECEC) staff are provided with pedagogical tools addressing healthy food exposure and child food acceptance. Objectives: a) evaluate the effectiveness of provision of the Nutrition Now resource on child diet and diet-related outcomes, with special attention to the influence of socio-economic position, b) gather information on the effectiveness of the implementation process to inform forthcoming scale-up and c) perform trial- and model-based economic evaluations. This is a hybrid type 1 implementation study, focusing on evaluation of effectiveness. A quasi-experimental design with pre- and post-tests, where one municipality gets access to the resource (n~800), while a matched non-equivalent control municipality (n~800) does not, will be used. Effectiveness will be assessed by examining e.g., diet outcomes, developmental outcomes, and feeding practices. The resource will be implemented in ECEC settings and made available to pregnant women and parents through the Norwegian system of maternal and child health (MCH) care. The implementation process includes iterative adjustments and implementation strategies from the implementation framework Expert Recommendations for Implementing Change (ERIC) informed by dialogues with stakeholders. Implementation outcomes (e.g., acceptability and adoption) will be assessed through questionnaires and interviews with parents, ECEC and MCH staff, with particular attention to ethnic diverse groups. Both within-trial and modelling-based economic evaluation will be performed. Nutrition Now will bridge the existing evidence-to-practice gap through rigorous scientific effectiveness evaluation of municipal scale up and inform subsequent county scale up. The study is the first to implement efficacious nutrition interventions in early life with potential for health improvement using technology to maximise the reach and impact of both parental and MCH dietary guidance and ECEC practice.

**Clinical Trial Registration:**

https://www.isrctn.com/, identified ISRCTN10694967.

## 1 Introduction

Research on the effectiveness of dietary interventions and their public health impact in real-world, high-income settings is scarce (1). Further, only a fraction of efficacious health interventions have been successfully scaled up and implemented in real life settings (2). This represents a clear evidence-to-practice gap, with a loss of opportunity to improve practice and an unnecessary waste of resources (3).

The first 1000 days of life, from conception until a child’s second birthday, is a window of opportunity for promoting long-term health and well-being (4). Failure to meet nutritional needs in this period is strongly linked to raised lifelong risk of obesity and non-communicable diseases (NCDs) (5). The importance of diet-related interventions in the first 1000 days can be ascribed to at least three mechanisms operating at different levels. First, unmet demands in this critical phase of early development can affect the size and structure of organs, increasing the risk of developing hypertension, cardiovascular disease, type 2 diabetes, and obesity (6). Extensive growth and neurodevelopment take place in this period, and optimal development depends on the amount and quality of food and nutrients provided (4, 7). Second, adverse nutritional conditions may permanently affect gene expression and program the body towards the development of NCDs (8). Third, dietary preferences and food habits are formed early in life, influenced by feeding practices of parents and others, the variety of foods offered (9), and the socioeconomic, cultural and educational context of the family (10). The public health significance of the first 1000 days is supported by cost-benefit and cost-effectiveness analyses showing large economic and social returns from early-life investment, especially from optimising nutrition (11).

The World Health Organization (WHO) promotes a lifecourse approach to promoting health and eradicating health inequality (12). A lifecourse approach to nutrition focuses on targeting nutrition during sensitive dietary transitions, such as the initiation and maintenance of breastfeeding, the introduction of complementary foods, and the transition to early childhood education and care (ECEC) when meals are eaten outside the home environment and shared with peers (13). These transitions need to be acknowledged and addressed through primary care and an education system, which aims to give parents and ECEC providers knowledge and skills to support their children to acquire dietary habits that promote rather than constrain their future health.

The Norwegian government sets goals to improve population diet and dietary habits in the Norwegian National Action Plan for a Healthier Diet (2017–21), which has since been extended to 2023 (14). The mid-term evaluation report, launched in June 2020, reveals that Norway is not on track to reach these goals and that much remains to be done in all age groups (15). One example is the large increase in the use of commercial baby food pouches, which rose from no usage in 2008 to 61% of 6 months old receiving them almost every day in 2020 (16, 17). These pouches are convenient, but their contents are often high in sugar (18, 19). Frequent use of such products limits texture and flavour learning, prevents use of healthy feeding practices such as responsive feeding, and increases the risk of childhood obesity (18). The inadequate and sometimes complete lack of vegetables served in ECEC settings is another example (20). Further, unacceptably large differences exist across socioeconomic groups regarding diet during pregnancy, infant and child feeding practices and overall diet quality (21, 22).

Dietary care is a complex part of child rearing. Hence, many parents seek out information on diet and feeding practices, some from questionable sources such as social media influencers (23, 24). There is a potential role for primary health services (Maternal and Child Health (MCH) care) to support parents to develop appropriate skills in feeding their child, regardless of background. The INFANT study demonstrated an untapped potential for integrating flexible, evidence-based e-learning resources to support in-person food-based dietary guidance (25). ECEC is another municipal service that is important for child diet, and which represents an opportunity to enhance meal interactions and diet quality. In Norway, most children from 1 to 5 years attend ECEC, including almost 90% of 1–2-year-olds (26). Throughout a child’s total time spent in ECEC an estimated 3000-4000 meals are eaten. Thus, ECEC offer an important setting for exposure to healthy foods; staff’s feeding style and practices may play a significant role in establishing early healthy eating habits in Norway’s children (27). Stakeholders at municipality level can support citizens’ adoption of a healthy diet early in life through strategies, prioritizing efforts and resources that enhance health and wellbeing in target groups.

**Figure 1:**
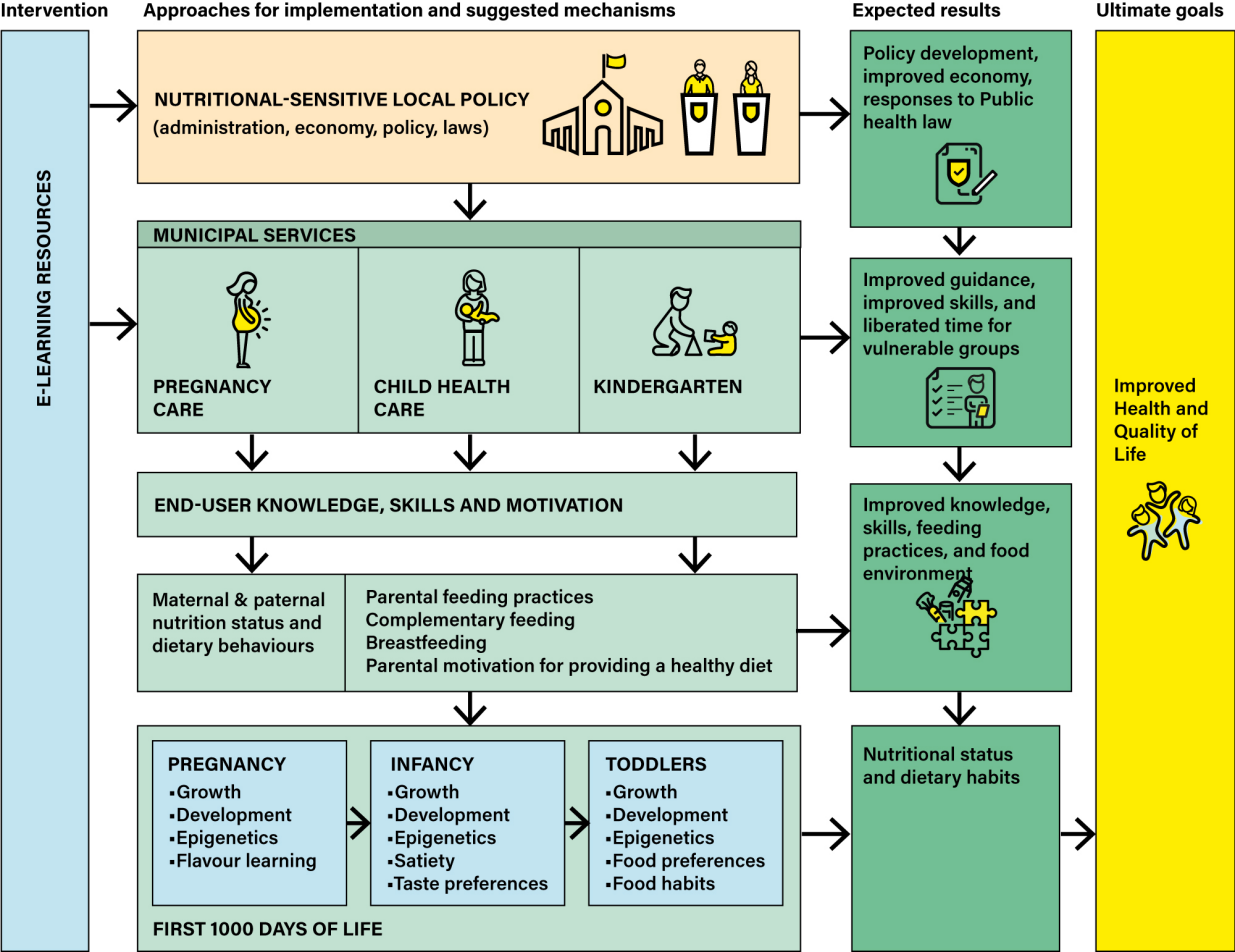
Logic model for Nutrition Now.

We have previously developed and reported on the efficacy of four interventions that target diet during pregnancy, parental feeding practices, and ECEC diet quality and meal environment, respectively. All four interventions have shown promising indications of dietary improvement (28–32) (**Additional File 1, Table S1**). These interventions are digital, largely video-based and grounded in social cognitive theory (33), addressing the interaction between person, environment, and behaviour. In the Nutrition Now project, we have combined these four interventions into one joint e-learning resource (the Nutrition Now resource) for parents, MCH staff and ECEC staff. The Nutrition Now resource will be implemented in one municipality through municipal services for MCH care (age: 0-2 years) and ECEC departments for children aged 1-3 years, as shown in **Figure 1**, which depicts hypothesised mechanisms to explain how the intervention may work. Increased knowledge, skills, and motivation are thought to improve dietary behaviors and feeding practices that will in turn affect pregnancy, infant and toddler health outcomes. We also aim for the implementation to influence local policy relating to physical and social aspects of the food environment. The ultimate goal of the Nutrition Now project is to reduce the burden of obesity and NCDs and improve health in quality of life in Norway through improved diet and diet-related behavior in the first 1000 days of life (**Figure 1**).

The Nutrition Now Project comprises two studies, with the results of the first informing the second. The field of dissemination and implementation research is constantly evolving. While the established standards of evidence for efficacy, effectiveness and scale-up research (34) have more distinct phases of effectiveness and implementation, newer hybrid designs propose to increase the speed of moving research into practice by blending the two stages of effectiveness evaluation and implementation, emphasising either effectiveness or implementation dependent on the type of hybrid study. The current study is a hybrid type 1 study [according to criteria from Curran et al. (35) and Landes et al. (36)] focused primarily on evaluating the effectiveness of providing access to the Nutrition Now resource, and secondly on evaluating implementation and cost-effectiveness. The subsequent study will be a hybrid type 3 study with a primary focus on implementation outcomes (35, 36). Scale up of the Nutrition Now resource to county level will be described in a later paper.

## 2 Methods/design

### 2.1 Aims and objectives

The aim of the study is to assess the impacts on child diet quality of implementing an e-learning resource (the Nutrition Now resource) comprising four digital interventions of known individual efficacy in a community setting.

The three main objectives are to a) evaluate the effectiveness of the Nutrition Now resource on outcomes related to diet and diet-related behaviors in early life, with special attention to the influence of socio-economic position b) observe and gather information on the implementation process to identify implementation strategies for use in a forthcoming scale up, with a special focus on ethnic minority groups and c) perform both trial- and model-based economic evaluations of the implemented Nutrition Now resource.

### 2.2 Outcomes

**Figure 2:**
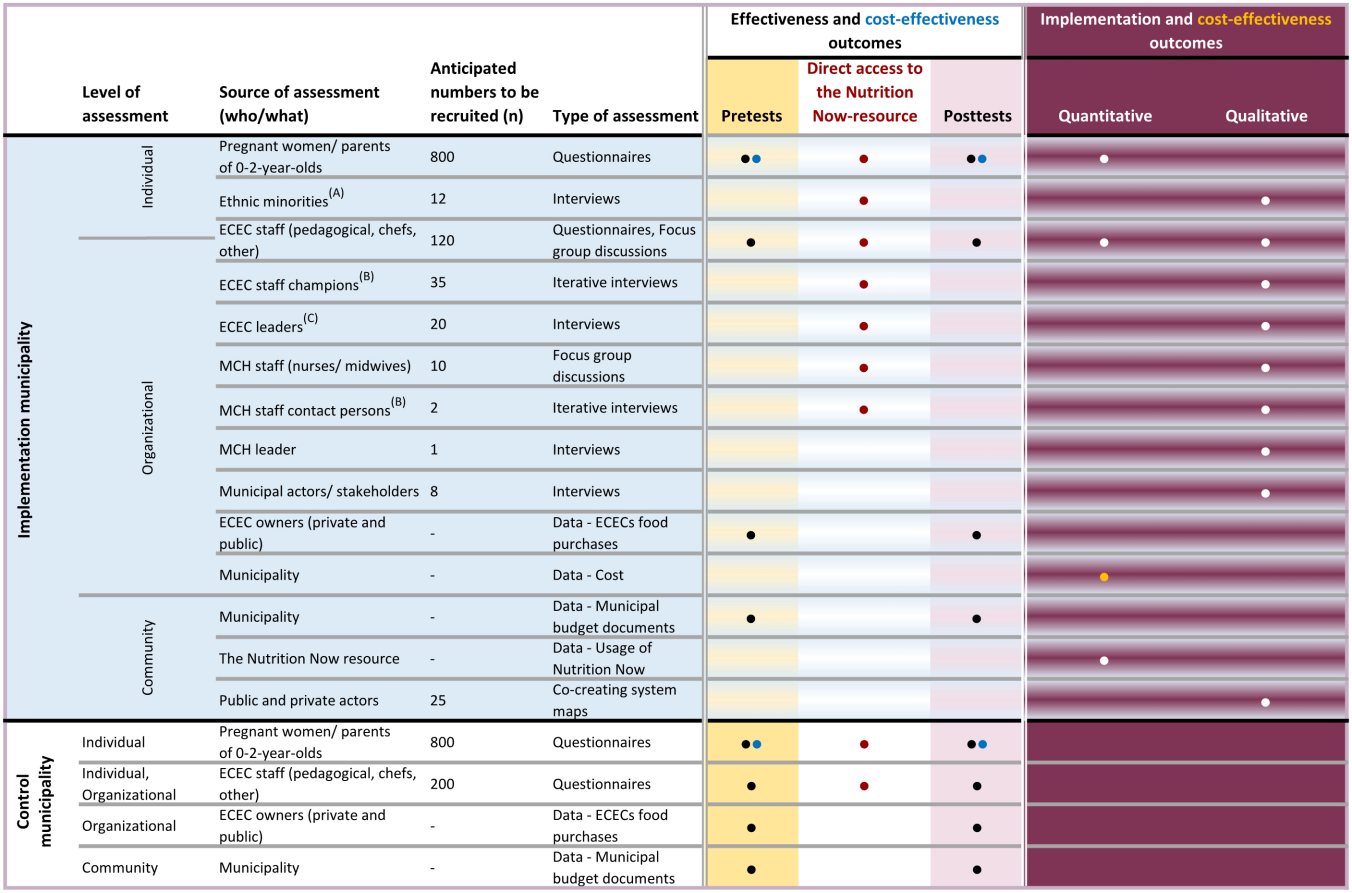
Overview of assessments, participants, and types of outcome measures in the mixed methods Hybrid 1 trial of the Nutrition Now project. **(A)** Ethnic minorities invited to participate in qualitative interviews in addition to the assessment that applies to all recruited pregnant women/parents of 0-2 year-olds. **(B)** ECEC staff champions and MCH staff contact persons are appointed staff members invited to participate in short iterative interviews. **(C)** ECEC leaders provide socio-demographic data for the effectiveness evaluation to be assessed at the ECEC level in addition to qualitative interviews.

The outcomes from this study are divided into three main categories: effectiveness outcomes, implementation outcomes, and outcomes from the economic evaluation (**Figure 2**). The effectiveness and implementation outcomes are measured on both the individual, organisational, and community level. An overview of all outcomes, the evaluation data relating to these outcomes, the level of measurement, and the instruments that will be used to collect the data is shown in **Table 1**.

**Table 1:** Effectiveness, implementation and cost-effectiveness outcomes in Nutrition Now.

Outcomes	Evaluation data	Data collection tool	Level
Effectiveness			
Aspects of child diet quality^1^	Frequency of intake of fruits, vegetables, pulses, and sugary drinks.	Short food frequency web-based questionnaire.	Individual
Breastfeeding^2^	Intention of breastfeeding, exclusive breastfeeding duration and any breastfeeding duration	Short web-based questionnaire	Individual
Child eating enjoyment^2^	Child Eating-enjoyment, reported by parents and ECEC staff	Child eating-enjoyment scale (37)	Individual
Child quality of life^2^	Pediatric Quality of Life Infant Scales	PedsQL (38) – Web-based questionnaire	Individual
Child development^2^	Developmental milestones	Ages and stages (18 months) ASQ3 (39)	Individual
Anthropometric measures (mother, child) ^2^	Weight change in pregnancy, mother’s weight and height, child’s weight and length	Short digital web-based questionnaire	Individual
Pregnancy complications	Preeclampsia, gestational diabetes, preterm delivery		Individual
Maternal diet in pregnancy^2^	Diet quality score	Diet screener, web-based questionnaire	Individual
Maternal quality of life^2^	Satisfaction with life score	SWLS - Satisfaction with life scale (40) – Web-based questionnaire	Individual
Parental meal- and feeding practices^2^	Role modelling, responsive feeding, family meal participation, meal distractions, frequency of home cooking, repeated exposure.	Short digital web-based questionnaire	Individual
ECEC staff meal- and feeding practices^2^	Role modelling, meal practices, repeated exposure, collaboration with parents.	Short digital web-based questionnaire	Individual/organizational
ECEC fruit and vegetable availability^2^	Fruit and vegetables purchases in ECEC	Receipts collected retrospectively, digital when available.	Organizational level
Municipal spend on nutrition ^2^	Resources (staff/money) allocated to nutrition or food-related activities over the annual budget in the municipality.	Publicly available political protocol documents. Collected retrospectively.	Community level
Implementation			
Acceptability	Whether Nutrition Now is liked, welcomed, appealing and approved	AIM-scale (41) Web-based questionnaire Interviews	Individual (a,b,d), and organizational (e,f,g)
Appropriateness	Whether Nutrition Now is fitting, suitable, applicable and a good match	IAM-scale (41) Web-based questionnaire Interviews	Individual (a,b,d), and organizational (e,f,g)
Feasibility	Whether Nutrition Now is implementable, possible, doable and easy to use	FIM-scale (41) Web-based questionnaire Interviews	Individual (a,b,d) and organizational (e,f,g)
Reach	Number of users (incl. ECEC) and characteristics of users	Data from website	Individual and organizational
Adoption/Uptake	Number of participants (incl ECEC)/number invited, what parts of the resources are used, and for how long.	Data from recruitment Interviews	Individual (a,b) and organizational (e,f,g)
Implementation/Fidelity	To what extent Nutrition Now is delivered as planned	RE-AIM.org (42) Interviews	Individual (a,b) and organizational (e,f,g)
Maintenance/Sustainability	To what extent Nutrition Now is still being used 7 and 12 months after implementation, how it is adapted.	RE-AIM.org (43) Interviews	Individual (a,b), organizational (e,f,g), community.
Cost-effectiveness			
Implementation costs	Costs of implementing the e-resource, including monitoring activities and management. Costs of the time spent by MCH and ECEC staff in using the resource.	Implementation costs/effectiveness outcomes	Organizational
Nutrition Now effectiveness outcomes	Quality of life score and developmental milestones.	PedsQL, Ages and stages (18 months) ASQ3 (39) in short digital questionnaire	Individual

^1^ Primary effectiveness outcome, ^2^ Secondary effectiveness outcome. Implementation outcomes will also be assessed in qualitative interviews. Level: Individual: ECEC staff (a), Health care center staff (nurses and midwifes) (b), pregnant women (c), parents of 0-24 months old children (d).

Organizational: ECEC leader (e), health care center leader (f), municipality (g), Community: network of public, voluntary and commercial stakeholders, including inhabitants in the municipality.

#### 2.2.1 Effectiveness outcomes

The primary effectiveness outcome is aspects of child diet quality, represented by intake of vegetables, fruits and berries, legumes/pulses and sugar sweetened beverages. Secondary outcomes include a) breastfeeding rates (including mixed feeding), b) child eating enjoyment c) health-related quality of life (infant), d) child development measures, e) self-reported anthropometrics (parental and child), f) maternal diet, g) maternal quality-of-life measures h) pregnancy complications, i) parental feeding practices, j) ECEC staff feeding practices k) ECEC fruit and vegetable availability and l) municipal spend on activities related to early childhood nutrition and feeding practices (see **Table 1**).

#### 2.2.2 Implementation outcomes

Seven outcomes will be used to evaluate the implementation process, including i) reach, ii) adoption, iii) implementation (fidelity), and iv) maintenance, as defined by RE-AIM (44, 45), and v) acceptability, vi) appropriateness and vii) feasibility, as defined by Proctor et al. (46) (**Table 1**). In addition, we will specifically assess adoption, acceptability, appropriateness, and feasibility in an ethnically diverse sample.

#### 2.2.3 Cost-effectiveness outcomes

The cost-effectiveness outcomes that will be evaluated are implementation costs in relation to the effectiveness outcomes child health-related quality of life and development measures.

### 2.3 Study design

In this hybrid type 1 mixed methods implementation study we use a quasi-experimental design. The CONSORT Extension for pragmatic trials with its checklist was used (47). A single intervention municipality will be given access to the Nutrition Now resource and another control municipality (non-equivalent control) will continue as normal (**Figure 2**). Data from participants resident in the intervention municipality will be collected in the form of a pretest before they are given access to the Nutrition Now resource. Subsequently post-test data will be collected until the child reaches 2 years of age (See Data collection). Participants in the control municipality will be asked to provide pre- and post-test data but will not receive access to the Nutrition Now resource. Additionally, a time-series design will be used for comparisons within the intervention municipality between groups of participants exposed to the Nutrition Now resource at different levels of duration, and different types of exposure (See Data collection).

Enrolment started September 2022. The anticipated recruitment period is from September 2022-to April 2023, and we envision that the last participant (if recruited in early pregnancy in April 2023) will complete the study in September 2025. The ECECs were recruited prior to September 2022 and MCH personnel and parents will be recruited autumn 2022.

Intervention municipality provision of access to the Nutrition Now resource starts upon enrollment in the study and is expected to continue beyond the study period as long as the municipality finds it useful.

### 2.4 Setting

The intervention and control municipalities are situated in different counties in coastal areas in the south of Norway. They have broadly comparable demographics: for example, they are comparable with respect to population size (appr. ~46000 inhabitants), number of births per year (~400), number of ECECs (54 vs 39) as well as education level, income, and family composition (**Table 2**).

**Table 2:** Comparability of intervention and control municipalities in the Nutrition Now project (data from 2017-2022).

Characteristics ^a^	Intervention (I)	Control (C)	Difference (I-C)
Population size (Q2, 2022) ^a^	45785	48024	-2239
Annual number of births (2021) ^a^	399	404	-5
**Population density (persons per km2) (2022) ^a^**	**178**	**62**	**116**
Immigrants and Norwegian-born to immigrant parents, % (2022) ^a^	15	15	0
Asia, Africa, Latin America, Oceania except Australia and New Zealand, and Europe except the EU/EEA/UK	9	8	0
The EU/EEA, United Kingdom, USA, Canada, Australia and New Zealand	7	7	0
The top three countries of origin for immigrants and Norwegian-born to immigrant parents	4	5	-1
#1 - Poland	2.0	2.9	-0.8
#2 - Syria	1.0	1.1	-0.1
#3 - Lithuania	0.9	1.1	-0.2
Persons per family/household (2022) ^a^	2	2	0
Children of single parents, % (2018-2020) ^b^	19	18	2
Higher educational level, people over 16y (1y+), % (2021) ^a^	33	29	4
Higher educational level (long), people over 16y (4y+), % (2021) ^a^	8	7	2
Not in employment or education, people over 45y, excluding retirees % (2018-2020) ^b^	23	20	3
Median income per family after taxes in NOK (2020) ^a^	519000	522000	-3000
Sustained low-income households, % (2017-19) ^b^	12	11	1
Number of Healthcare centers (2022) ^c^	1	6	-5
**Number of ECEC (2022) ^c^**	**54**	**39**	**15**
**Private ECEC, % (2022) ^c^**	**81**	**54**	**28**
Children 1-2 years in ECEC, % (2020) ^a^	85	82	3
Children in municipal ECECs who receive special educational assistance, % (2020) ^a^	6	3	3

^a^Data from Statistics Norway (48, 49).

^b^Data from the Norwegian Institute of Public health (50).

^c^Data from the official homepages of the municipalities (51, 52). Bold numbers show characteristics where there are large discrepancies between the intervention and control municipalities.

### 2.5 Study participant groups and recruitment

The participant groups in this project are listed in **Figure 2**. All pregnant women and parents of 0-2-year-olds living in the intervention or control municipality are eligible for this study. For the sake of simplicity, we will refer to guardians/care givers/parents as parents although all are included in this notion. Further, all MCH nurses and midwives and ECEC leaders working in the intervention or control municipality, staff in ECECs in which the leaders have consented to participate, plus all other relevant municipality staff within public health in the intervention municipality are eligible to participate.

Pregnant women and parents of children aged 0-2 years will be recruited at 1) routine visits to the MCH centre by MCH nurses and midwives, 2) the municipality website and 3) the relevant ECEC’s website, with all routes leading to a registration website where participants are able to give digital consent. We have previously successfully recruited through MCH centres in a similar way (53). Persons from ethnic minority groups will be recruited for participation in in-depth-interviews, representing pregnant women, parents of 0–2-year-olds and parents of 1-2-year-olds in ECEC, *via* Non-Governmental Organizations and other arenas in the municipality using snowball sampling. ECEC leaders will consent on behalf of their ECEC and will then recruit team leaders at each unit and their staff by forwarding digital invitations to participate in the study. Midwives and public health nurses at the participating MCH centres and municipality stakeholders (public health workers, leaders) will be recruited by digital invitations from the research group.

The sample size in this study is capped by the annual birth rate in the two municipalities (~400 per group, e.g., a total of approx. 800 births each year). The primary effectiveness outcome, aspects child diet quality, exemplified by vegetable intake was used to check that the study would be sufficiently powered. In a previous efficacy trial of 298 1–2-year-olds, we observed a difference in the change from baseline to 6-month follow-up of +0.46 vegetable items per day in favour of the intervention group (n=148). Assuming a participation rate in this study between 50 and 80%, we anticipate a sample size of 510-820 from each municipality, of whom 100-160 will have potentially been exposed to all parts of the Nutrition Now resource (*in utero* to 2-years of age). The worst-case scenario, with a 50% participation rate and 100 participants being exposed to all parts of the Nutrition Now resource, would enable us to detect a difference in vegetable of intake between groups of 0.5 items per day (alpha of 0.05 and 80% power). We anticipate that several hundred participants will be exposed to the intervention to some extend enabling other comparisons between the intervention and control municipality, with larger participant numbers.

### 2.6 The Nutrition Now resource

**Figure 3:**
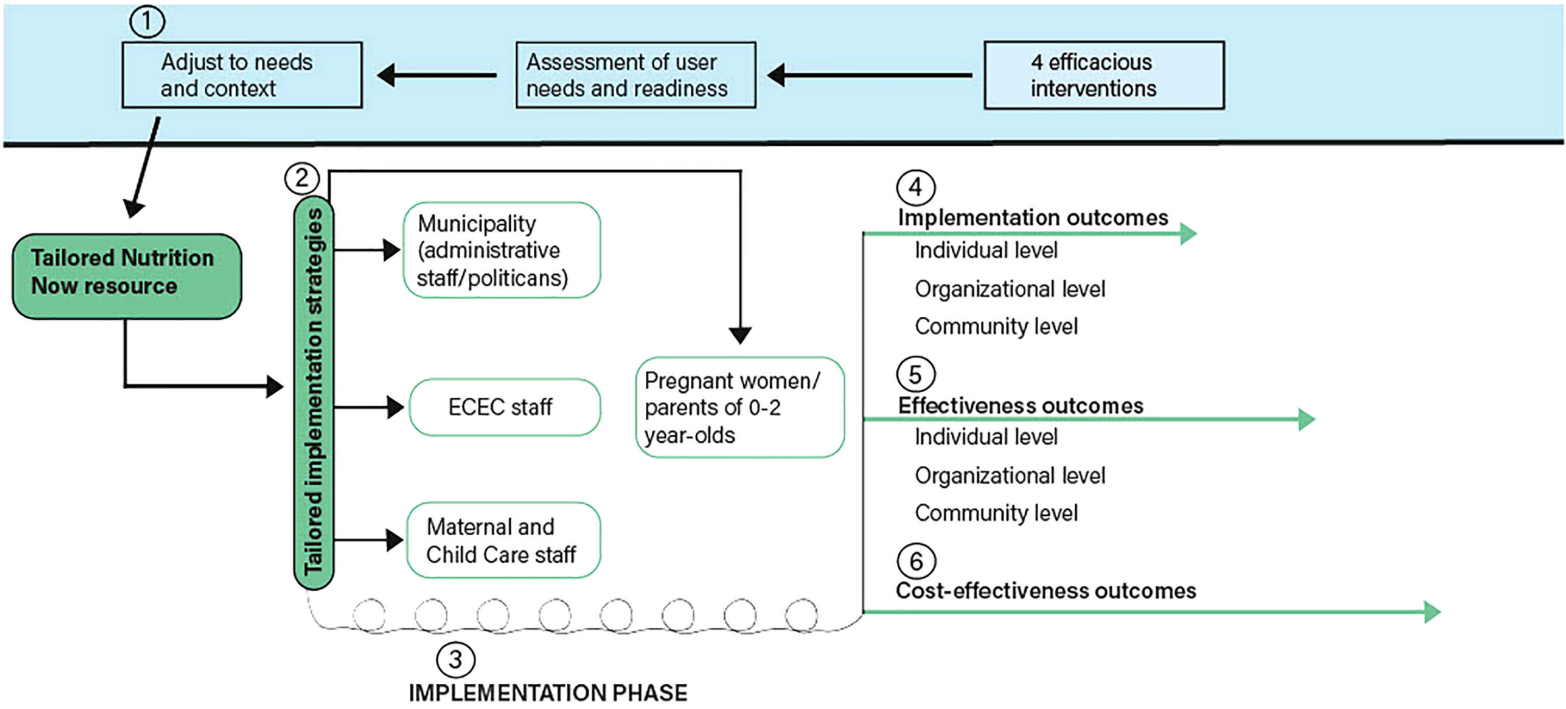
DIEM in the Nutrition Now project.

The Nutrition Now resource comprises four efficacious e-learning interventions (details including theoretical underpinning provided in **Additional File 1, Table S1**) offered through a website targeting different transitions early in life, from pregnancy to child age 2. The content and structure of the original interventions has been modified and adapted to context and user needs (**Figure 3**) in the relevant settings for this study (family-, MCH centre- and ECEC- settings). The modification also includes offering the Nutrition Now resource in English and Arabic languages in addition to the Norwegian version, as requested from both MCH and ECEC staff (on behalf of parents). Results from other adjustments in the development of the Nutrition Now resource will be described and published in detail elsewhere.

The Nutrition Now includes topics presented as core components addressing 1) the importance of diet early in life, 2) promotion of breastfeeding, 3), the importance of parental role in food provision and shaping child diet, 4) responsive feeding, 5) shared meals, 6) knowledge and experience of food preparation, 7) sensory play with vegetables, and 8) ECEC collaboration with parents (**Additional File 2, Table S2**). These topics are presented by videos, graphics, images and “easy to read” texts.

### 2.7 Project procedures in implementing the Nutrition Now resource

The process of implementing the Nutrition Now resource in the intervention municipality is guided by the Dynamic Integrated Evaluation Model (DIEM), the development of which was led by one of the co-authors (UvTS) (54). We will use the iterative implementation process as proposed in DIEM, in which the planned implementation strategies can be adapted continuously based on collected data during the implementation process. The iterative process builds on employing rapid improvement cycles in a participatory approach that ensures a continuous process of changes. From a practice perspective, self-reflection and continuous development are important, and short, iterative interviews with stakeholders (ECEC and MCH) will serve as support for this (54). **Figure 3** shows elements from DIEM alongside the specific elements of the current study.

As shown in **Figure 3**, the tailored Nutrition Now resource is delivered through implementation strategies (2), already adjusted to needs and context (1) for use in the project’s iterative implementation phase. This enables further adjustments of the strategies as the project is rolled out and yields implementation outcomes continuously (3). Different implementation strategies target MCH staff, ECEC staff, the municipality level, and pregnant women and parents (**Figure 3**, **Tables 3**–**5**).

**Table 3:** Implementation strategies targeting Nutrition Now implementation delivered through Municipality (Organizational level).

Category (55, 56)	Strategy (# denotes ERIC implementation strategy) (57)	The Actor *(deliver strategy)*	The Action *(specific action, steps or process)*	Action target *(affected by strategy)*	Implementation outcome(s)	Temporality and Dose	Justification
**Use evaluative and iterative strategies**	#4 Assess for readiness and identify barriers and facilitators	Implementation researchers	Meetings with stakeholders in municipality	Stakeholders in municipality	Adoption Appropriateness Feasibility	Pre-implementation from project start until the end of September 2022	Identify factors that may disrupt implementation, and strengths that can be exploited in the implementation effort
	#56 Purposefully reexamine the implementation	Implementation researchers	Conduct monthly short status meetings with public health coordinator	Public health coordinator	Feasibility	Monthly - during the pre-implementation and implementation process	Anchor implementation strategies, optimize implementation strategies to maximize intervention effectiveness
**Develop stakeholder interrelationships**	#6 Build a coalition	Implementation researchers	Conduct meetings with stakeholders	Stakeholders in municipality	Feasibility Fidelity	Pre implementation and during implementation	Ensure a good collaborative environment between stakeholders and research team at the municipality level
	#17 Conduct local consensus discussions	Implementation researchers	Conduct Mapping session	Stakeholders in municipality and politicians	Feasibility Sustainability	Pre-implementation, one time Amounts to 2 hours spent by each stakeholder/politician at municipality level	Establish a consensus for a need to improvement of early childhood nutrition in the municipality and the need for implementing Nutrition Now
	#47 Obtain formal commitments	University and municipality leaders	Written formal agreement between municipality and University	Municipality	Feasibility Sustainability	Pre implementation	Ensure key partners to commit to the signed agreement
**Engage consumers**	#Use mass media	Implementation researchers and stakeholders in municipality	Interviews in Newspapers, municipality website, blogpost	Target population in municipality	Adoption Reach	Pre implementation (newspaper interviews) and during implementation (website and blogpost)	Contribute to make Nutrition Now known and familiar to the target population
**Change infrastructure**	# Mandate change	Stakeholders in municipality	Declaration of priority given to Nutrition Now	MCH and ECEC in municipality	Adoption Fidelity Sustainability	Pre implementation and implementation - ongoing	Establish a determination to implement Nutrition Now

**Table 4:** Implementation strategies targeting Nutrition Now implementation delivered through Maternal and Child Health (MCH) care center.

Category (55, 56)	Strategy (# denotes ERIC implementation strategy) (57)	The Actor *(deliver strategy)*	The Action *(specific action, steps, or process)*	Action target *(affected by strategy)*	Implementation outcome(s)	Temporality and Dose	Justification
**Use evaluative and iterative strategies**	#4 Assess for readiness and identify barriers and facilitators	Implementation researchers	Conduct in-depth interviews with 6 in 12 MCH nurses and one focus group discussion (FGD) with all 5 MCH midwives.	MCH nurse and midwives	Adoption Appropriateness Feasibility	Pre-implementation from project start until the end of September 2022 One in-depth interview and one FGD	Identify factors that may disrupt implementation, and strengths that can be exploited in the implementation effort
	#18 Conduct local needs assessment	Implementation researchers	Conduct in-depth interviews with 6 in 12 MCH nurses and one focus group discussion (FGD) with all 5 MCH midwives.	MCH nurse and midwives	Appropriateness Feasibility	Pre-implementation One in-depth interview and one FGD	Use the experiences and key knowledge the partners have regarding the target groups in order to tailor intervention to context
	#56 Purposefully reexamine the implementation	Implementation researcher	Conduct monthly short iterative interviews with one selected MCH nurse and midwife, respectively.	MCH nurse and midwives	Feasibility	During the implementation process (first six months) Six short interviews with staff	Optimize implementation strategies to maximize intervention effectiveness
**Adapt and tailor to context**	#51 Promote adaptability	Implementation researchers	Tailor the intervention to local context, e.g., by focusing on easy-to-understand texts, extensive use of visual and humorous elements as well as translating e-learning resource into English and Arabic language.	Pregnant women and parents of infants and toddlers	Acceptability Appropriateness Feasibility	Pre-implementation Dosage amounts to six months for 6 employees	Ensure that the Nutrition Now e-learning resource is suited/adapted for the target groups and context
	#63 Tailor strategies	Implementation researchers in collaboration with web designer	Develop ways to convey information about the e-learning resource considered as appropriate by MCH nurses and midwives, e.g., small information cards in three languages, posters at MCH center, instructional pamphlet targeting MCH staff.	Pregnant women and parents of infants and toddlers	Acceptability Appropriateness Feasibility	Ongoing during implementation Dosage amounts to one week of work	Tailor implementation strategies and leverage enablers identified.
**Develop stakeholder interrelationships**	#6 Build a coalition	Implementation researchers	Participate in internal educational meeting for MCH nurses. Arranged 3 + 3 workshops with MCH nurses and midwives, respectively.	MCH nurse and midwives	Acceptability Feasibility	Pre implementation until e-learning resource is distributed Amounts to 20 hours spent by MCH staff and 3 researchers	Ensure a good collaborative environment between stakeholders at the setting level
	#17 Conduct local consensus discussions	Implementation researchers	Conduct repeated workshops with MCH nurses and midwives separately (3 + 3) having discussions about e-learning resource suitability and content priorities.	MCH nurse and midwives	Appropriateness Feasibility	Pre-implementation Amounts to 20 hours spent by MCH staff and 3 researchers	Agree upon resource content deemed relevant for the target groups
	#38 Inform local opinion leaders	Implementation researchers	Conduct initial and repeated preparatory meetings with MCH service leaders.	MCH service leaders	Feasibility	Three meetings, in total 3 hours spent by staff and researchers	Anchor implementation strategies with service leaders to avoid misconceptions
**Train and educate stakeholders**	#15 Conduct educational meetings	Implementation researchers	Conduct educational meetings with MCH nurses, midwives and service leaders to teach them about the Nutrition Now innovation (the e-learning resource)	MCH nurse and midwives	Acceptability	Pre implementation and at the start of the implementation. Amounts to 1 hour spent by staff	Increase partners’ key knowledge about Nutrition Now resource content and project fundamentals
	#29 Develop educational materials	Implementation researchers in collaboration with web designer	Develop video and supporting written material to support staff: A short manual for MCH nurses and midwives briefly explaining how to convey the content and use of the e-learning resource to parents. Small information cards with QR codes leading parents directly to the e-learning resource. Educational videos explaining rationale and how to use the resource	MCH nurse and midwives Pregnant women and parents of infants and toddlers	Feasibility	Pre implementation phase 1. Post phase 1 to develop for phase 2. 3 days of work for two researchers and 2 days for web-designer	Need for instructional materials that make it easier for staff to convey the Nutrition Now resource and for pregnant women and parents to learn about how to use the resource
	#31 Distribute educational materials	Implementation researchers	Distribute educational material in person in connection with educational meetings	MCH nurse and midwives	Fidelity Feasibility	Pre-implementation. 2 days of work for 2 researchers	Provide educational materials to staff to facilitate delivering the intervention
**Support clinicians**	#58 Remind clinicians (including end-users of the e-learning resource)	Implementation researchers	Conduct follow-up meetings every six months Reminder e-mails with new resource content at regular intervals	MCH nurse and midwives Pregnant women and parents of infants and toddlers	Adoption Sustainability	Throughout implementation One day work for two researchers	Help MCH staff to recall information and/or prompt them to use the e-learning resource.

**Table 5:** Implementation strategies targeting Nutrition Now implementation delivered through Early Childhood Education and Care (ECEC).

Category (55, 56)	Strategy (# denotes ERIC implementation strategy) (57)	The Actor *(deliver strategy)*	The Action *(specific action, steps or process)*	Action target *(affected by strategy)*	Implementation outcome(s)	Temporality and Dose	Justification
**Use evaluative and iterative strategies ** ** **	#4 Assess for readiness and identify barriers and facilitators	Implementation researchers	Conduct individual interviews with 12 ECEC staff and two focus group interviews Conducted meetings with ECEC heads	ECEC staff	Adoption Appropriateness Feasibility	Pre-implementation from project start until the end of September 2022 12 interviews and 2 focus group interviews	Identify factors that may disrupt implementation, and strengths that can be exploited in the implementation effort
** **	#18 Conduct local needs assessment	Implementation researchers	Conduct individual interviews with ECEC staff to assess needs	ECEC staff	Appropriateness Feasibility	Pre-implementation 12 individual interview and 2 focus group interviews	Use the experiences and key knowledge the ECEC staff have regarding the setting in order to tailor intervention to context
	#14 Conduct cyclical small tests of change	Implementation researcher	Conduct short iterative interviews with one selected champion at each ECEC unit	Champion at ECEC	Appropriateness Feasibility	During the implementation process (first six months) Every third week, 5-7 minutes each time	Optimize implementation strategies to maximize intervention effectiveness
	# 27 Develop and organize quality monitoring systems	Implementation researcher	Monitor implementation process through iterative interviews	Champion at ECEC	Adoption Feasability	During the implementation process (first six months) Every third week, 5-7 minutes each time	Use the input from champions to ensure quality and improvement of intervention strategies
	# 46 Obtain and use patients/consumers and family feedback	ECEC staff	Use advice in e-learning resource regarding parental collaboration and get feedback from parents	ECEC parents	Appropriatedness		Parental feedback to motivate further implementation by ECEC staff
**Provide interactive** **assistance**	# 33 Facilitation	Implementation researcher	Supportive researcher available for problem solving and support interpersonal relationship	ECEC Staff, ECEC champions	Feasability Fidelity	Ongoing during implementation	Ensure a positive collaborative environment between ECEC stakeholders at the setting level
**Adapt and tailor to context **	#63 Tailor strategies	Implementation researchers in collaboration with web designer	Develop ways to convey information about the e-learning resource considered appropriate by ECEC staff	ECEC staff	Acceptability Appropriateness Feasibility	Ongoing during implementation	Tailor implementation strategies and leverage enablers identified
**Develop stakeholder interrelationships **	#6 Build a coalition	Implementation researchers ECEC leaders Champion	ECEC leaders and champions participated in educational meeting ECEC leaders support champions, through e-mails and meetings Champions are encouraged to build relations with colleagues, through newsletters and the Nutrition Now resource	ECEC leaders and champions	Acceptability Feasibility	Pre implementation until e-learning resource is distributed Four meetings with ECEC leaders prior to implementation	Ensure a positive collaborative environment between ECEC stakeholders at the setting level
** **	#35 Identify and prepare champions	ECEC leaders and reseachers	Select a champion at each ECEC and clarify understanding of role as an implementation agent	Champion at ECEC	Adoption Acceptability Appropriateness Feasibility Fidelity	Pre implementation	Anchor and ensure the implementation at each ECEC unit
**Train and educate stakeholders **	#15 Conduct educational meetings	Implementation researchers	Conduct educational meetings with ECEC leaders prior to implementation about the importance of early childhood nutrition and seminars during implementation about how Nutrition Now is implemented in different ECECs	ECEC leaders and Champion	Acceptability Fidelity	Pre implementation and 2 seminars during the first 6 months of implementation. ECEC leaders: 4-5 hours spent by each, Champions: 1-2 hours	Increase partners’ key knowledge about Nutrition Now resource content and how it can be adopted
** **	#29 Develop educational materials	Implementation researchers in collaboration with web designer	Develop video and supporting written material (e-mails and messages within the e-learning resource) to support ECEC staff. Educational videos explaining rationale and how to use the resource. Posters to put up in the ECEC units	Champion and ECEC staff	Feasibility	Pre implementation	Need for instructional materials that make it easier for ECEC staff to convey the Nutrition Now resource and how to best use the resource
** **	#31 Distribute educational materials	Implementation researchers	Distribute educational material digitally and by mail	ECEC leaders and champion	Fidelity Feasibility	Pre implementation	Provide educational materials to staff to facilitate delivering the intervention
**Support clinicians **	#58 Remind clinicians	Implementation researchers	Reminder e-mails with new resource content at regular intervals and digital newsletters with support on how to progress with the intervention including change processes among ECEC staff	Champions at ECEC	Adoption Sustainability	During implementation	Help ECEC staff to recall information and/or prompt them to use the e-learning resource
**Engage consumers**	# 41 Involve patients/consumers and family members	ECEC leaders	Post URL to parent part of the e-learning resource on ECEC website	ECEC parents	Feasability Adoption	Start of the implementation, one time	Create synergy that enhance ECEC staff’s motivation to follow up the implementation
**Change Infrastructure**	# 44 Mandate change	ECEC champions	ECEC champion lead and holds meetings with ECEC staff regularly	ECEC staff	Adoption Feasability Sustainability	During implementation	Create structures (e.g., staff meetings) that enhance the implementation

#### 2.7.1 Selection of implementation strategies

Assessing barriers and facilitators to implementation was used in selecting the implementation strategies. A pragmatic approach was used building on previous experience and dialogue with MCH and ECEC staff, considering and balancing feasibility, impact, and cost. Moreover, all implementation strategies are in line with those deemed feasible and important by the ERIC-framework (Expert Recommendations for Implementing Change) (57) and adjusted to context (57) (**Tables 3**–**5**). The implementation strategies and their operationalization and justification are presented in **Tables 3**–**5**. The strategies are grouped according to the nine categories or clusters developed through a stakeholder participatory approach in the ERIC-study (55).

#### 2.7.2 Implementation strategies targeting organizational level

We will employ a set of seven implementation strategies to target the political and administrative leadership level in the municipality, including assessing barriers and facilitators to implementation, local consensus discussions and coalition building. Close collaboration with the municipal public health coordinator is considered essential for implementation. A co-creation process with stakeholders in the municipality including politicians resulted in a systems-map reflecting what they as a group consider the main determinants of child diet. A pre-existing formal agreement between the University and the intervention municipality facilitated access to the different settings (**Table 3**).

#### 2.7.3 Implementation strategies targeting parents and MCH staff (individual level)

We have used three strategies targeting pregnant women and parents directly, all of which relate to tailoring and promoting adaptability of the Nutrition Now resource (**Table 4**). All texts and videos are provided in English and Arabic in addition to Norwegian to reach culturally diverse groups. Regular reminders with emails linking to age specific topic will be sent to the parents to promote use of the Nutrition Now resource.

We also employ a set of implementation strategies targeting MCH staff (**Table 4**) within the categories “use evaluative and iterative strategies”, “adapt and tailor to context”, “develop stakeholder interrelationships”, “train and educate stakeholder” and “support clinicians”.

#### 2.7.4 Implementation strategies targeting ECEC staff

We will utilize a set of implementation strategies targeting ECECs (**Table 5**). These include the categories “use evaluative and iterative strategies”, “provide interactive assistance”, “adapt and tailor to context”, “develop stakeholder interrelationships”, “train and educate stakeholder”, “support clinicians”, “engage consumers” and “change infrastructure”. Identifying team leaders at each ECEC unit is regarded as a crucial strategy as they are important implementation champions to oversee that implementation takes place. They will also participate in short iterative interviews to closely monitor the implementation process.

### 2.8 Data collection

Quantitative and qualitative data will be collected in the different parts of this study (**Figure 2** and **Table 1**).

*Quantitative data* for the effectiveness evaluation will be generated from questionnaires and objective measures. Questionnaire data will be obtained from i) pregnant women and parents of 0-2-years-olds, assessed during pregnancy, at child ages 6 weeks, 6 months, 12 months, 18 months, 24 months and ii) ECEC staff, assessed at baseline (pretest) and after 7 months (posttest).

**Figure 4:**
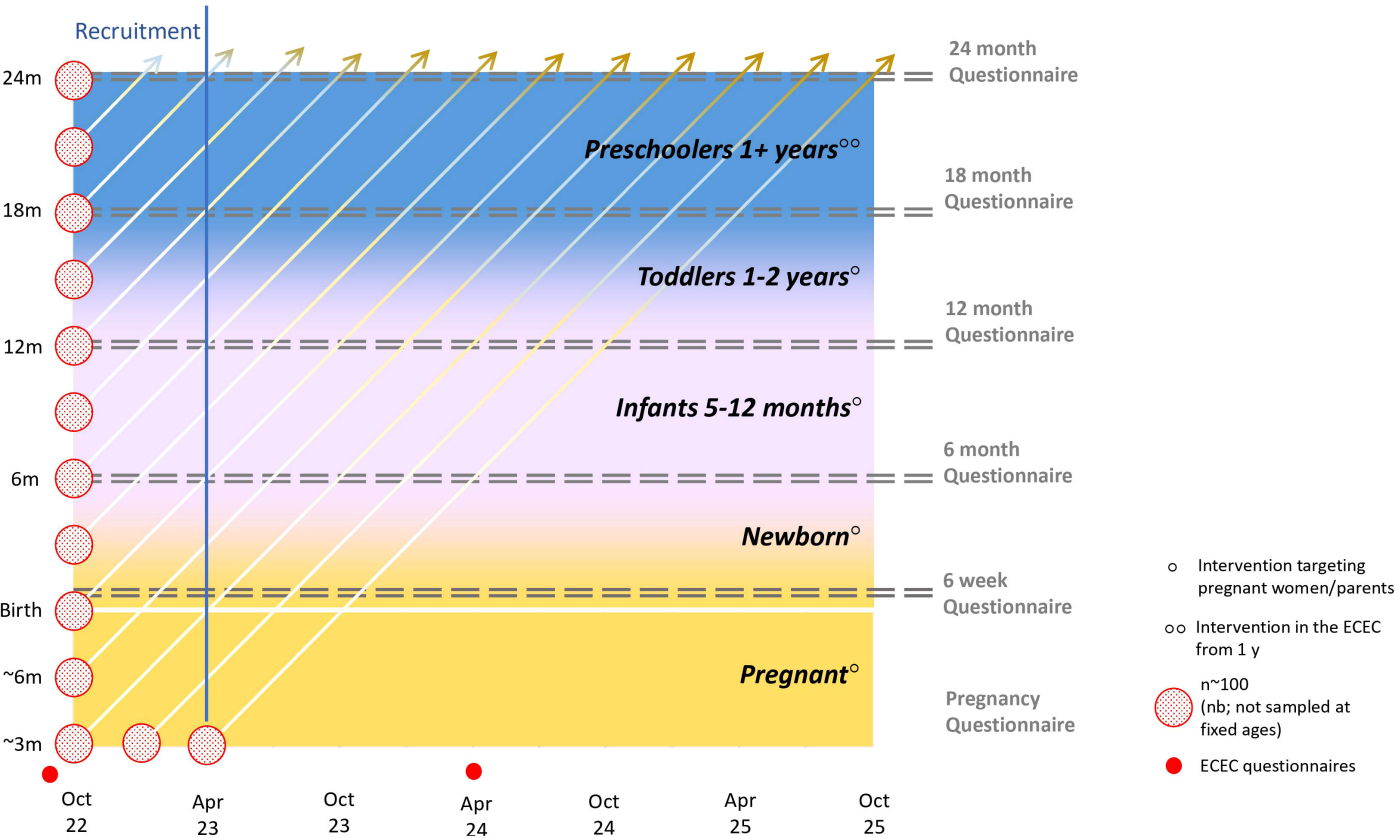
Overview of timeline for data collection, and exposure to the Nutrition Now resource (consisting of e-learning resources targeting different phases in a child’s 1000 first days) in the intervention municipality in the effectiveness trial. Pregnant women and parents of children between 0-2 years of age will be recruited from October 2022 until April 2023 through the MCH centre route. Participants will be exposed to age-appropriate content of the Nutrition Now resource and will be asked to respond to successive age-specific digital questionnaires depending on whether they are pregnant or the child’s age. Participants are given access to the Nutrition Now resource after completing the first questionnaire (pretest). ECEC staff will be recruited and complete the pretest questionnaire in September 2022, before getting access to the Nutrition Now-recourse. The post-test ECEC-staff questionnaire will be distributed seven months into the implementation of the intervention.

**Figure 4** provides an overview of the timeline for data collection for effectiveness outcomes for the parental part of the study, and duration of exposure to the Nutrition Now resource according to point of inclusion in the intervention municipality.

Consenting pregnant women (at any time during pregnancy), and parents of children between 0-2 years (at any time in this age interval) will first be asked to complete a short digital questionnaire to provide socio-demographic data in addition to expected due date (for pregnant women), or child’s age (for parents). Thereafter they will be asked to complete a second questionnaire tailored to the age of the child. Access to the Nutrition Now resource will be provided when the age-specific questionnaire is completed. The dose and duration of exposure to the Nutrition Now resource will differ between the participants depending on when they enter the study. Only those who are pregnant when entering the study between October 2022 and April 2023 will potentially be exposed to the whole range of intervention content, whereas a 1-year-old whose parent(s) enter the study in the same period, will be exposed to the “Toddlers 1-2 years” component and, if in ECEC, also the “Preschoolers 1+ years” component (**Figure 4**). Two-year-olds recruited at the regular health visit will be given access to the “Toddlers 1-2 years” component after completing the 24 months questionnaire.

ECEC leaders and staff will respond to questionnaires when consenting to participate in the study and seven months after getting access to the Nutrition Now resource.

Participants (both parents and ECEC staff) recruited in the control municipality will be asked to complete the same questionnaires as intervention participants but will not have access to the Nutrition Now resource.

Data on food purchase history in the ECECs will be collected from both the ECECs in the intervention and control municipality, for the years 2022 and 2023, to obtain data on fruits and vegetables purchases. Municipal budget data for 2022 and 2023 will be collected to gather info on municipal spend on activities related to early childhood nutrition and feeding practices in both the intervention and control municipality.

*Quantitative and qualitative data will be collected to evaluate the implementation process and outcomes*. Quantitative data from parents and ECEC staff at seven and 12 months after being enrolled in the study, will be collected using questionnaires with items from the seven implementation outcomes described in **Table 1**. Additional data will be collected using checklists during iterative interviews with ECEC and MCH during the first six months of the project, and usage data will be collected from the Nutrition Now resource. Evaluation of acceptability, appropriateness, and feasibility of the Nutrition Now resource will be done using a validated and generic questionnaire developed by Weiner et al. (41).

Qualitative data will be collected from short iterative interviews with team leaders in ECEC and contact persons for MCH nurses and midwifes, to promote self-reflection and continuous development. Further, focus group discussions with ECEC and MCH staff will be conducted approximately seven months after start of the implementation period to explore experiences with the implementation strategies. Individual interviews with ECEC leaders and stakeholders at municipality level will also be conducted (**Table 1**).

In depth description of what motivates and/or hinders use of the Nutrition Now resource will be explored in ethnic minority groups to render the next version of the resource more inclusive. With an anthropological way of interviewing, multi-stage, in-depth, open-ended and ethnographic interviews will be conducted with sub-samples of pregnant women and parents within ethnic minority groups in the intervention municipality to understand how the implementation of the resource works in and affects their everyday life.

*Costs related to Nutrition Now resource development, maintenance and implementation* will be collected throughout the implementation period using a bottom-up, micro-costing approach which will involve direct enumeration and costing of each intervention input (58).

### 2.9 Analysis

#### 2.9.1 Effectiveness

Evaluating our effectiveness objective requires following a treatment policy estimand strategy (59, 60)- that is the effect of access to the Nutrition Now resource regardless of post-enrolment or intercurrent events such as non-compliance or discontinuation. This necessitates that outcome data be collected regardless of level of engagement and assumes that missing data are missing not at random (MNAR). Statistical models appropriate for de facto estimands will thus be used and the effect of MNAR on our effectiveness estimates will be assessed using sensitivity analyses. Confounding due to the non-randomised nature of the study, e.g., household size and income, parental education, ECEC status (public or private), will be assessed and adjusted for using regression models.

The main comparison will be between children and parents given access to all Nutrition Now components and those in the control municipality. Similar estimators of the Nutrition Now effectiveness will be available from comparisons within the intervention municipality since we will have data on approximately 100 parent-offspring pairs completely unexposed to the Nutrition Now resource as a result of the recruitment design (described above). The latter comparisons will improve causal inference by triangulating the evidence from two designs with different sources of bias (61). In this example they act as a sensitivity analysis to assess bias from residual confounding.

The recruitment strategy also creates variation in the number of components of the Nutrition Now resource each observational unit will be exposed to. Combined with the serial data collection points (pregnancy, 6 weeks, 6, 12, 18 and 24 months), we will be able to assess the effectiveness of each component of the resource in addition to duration of access.

#### 2.9.2 Implementation

*Quantitative:* We will report descriptive statistics for implementation outcomes assessed quantitatively [acceptability, appropriateness, feasibility, adoption and reach (**Table 1**)]. In addition, the number of people accessing the webpage, and sub-pages, and the time spent there, will be automatically registered in the Nutrition Now-resource. *Qualitative* interview data will be transcribed verbatim and coded deductively informed by the CFIR framework (62) and inductively to capture issues within and outside of this framework. We will use these findings to inform future scale-up at county level.

#### 2.9.3 Economic evaluation

We will conduct economic evaluation in two ways. First, we will perform a within-trial economic analysis to evaluate the cost-effectiveness of the trial, i.e., trial-specific intervention costs relative to intervention effect on the outcomes selected for the economic evaluation. The within-trial analysis will be a cost–utility analysis (CUA), estimating the incremental cost per quality-adjusted life years (QALYs) gained from being given access to the Nutrition Now resource. QALYs will be generated *via* measurement of utility values based on the Pediatric Quality of Life Inventory (PedsQL) Infant Scales (13-24 months) (38), a pediatric generic health-related quality of life (HRQoL) instrument. Additionally, cost-effectiveness analysis (CEA) will be performed by calculating incremental cost per unit developmental improvement measured with the widely used developmental screening tool, Ages & Stages Questionnaire (ASQ), validated for use in the Norwegian population (39, 63) (**Additional File 2, Table S2**).

Second, a decision analytic model will be used to model the potential long-term cost-effectiveness of the Nutrition Now intervention over a lifetime horizon. We will develop a birth cohort simulation model based on estimated exposure-outcome associations in the Norwegian Mother, Father and Child Cohort (64) to explicitly model a set of causal pathways known to link child development to diverse later life outcomes. The model will provide a framework for integrating effectiveness measures from the current study in addition to data from external studies, drawn from targeted literature searches. The decision analytic modelling will adopt a wider societal perspective.

### 2.10 Data management

A data management plan (DMP) has been developed and will be published elsewhere. The DMP is an ongoing document throughout the project period and is in accordance with Norwegian Research Council’s (NRC) guidelines. We plan to share anonymized data in the UiA data repository Dataverse. This will be done no later than upon acceptance for publication of the main findings from the final dataset. We will retain our data for five years after data collection has stopped. Hence, our data will be made open access in line with the NRC’s guidelines, prior to three years after the completion of the study. Standard meta-information about the data will be uploaded.

Data from the pregnant women and parents of 0–2-year-olds will be collected using digital questionnaires created and distributed electronically using an encrypted version of the web-tool “Nettskjema” linked to a secure server, the Service for Sensitive Data (TSD, in Norwegian, “Tjeneste for Sensitive Data”). TSD is constructed for storing and processing data in agreement with the Norwegian Personal Data Act and Health Research Act (65). Digital questionnaires created in “Nettskjema” will also be used to collect data from leaders and other staff in the ECEC.

## 3 Discussion

There are several potential challenges the project may face. Recruitment of participants and their continued participation is crucial for both the implementation and effectiveness part of this study. To reduce participant burden and maximise retention, we will keep the questionnaires short, easily comprehensible, and easy to complete. To avoid missing all data in the case of partly completed questionnaires, it will be possible to respond to only a few initial core questions, and still participate. Further, selection bias at recruitment between intervention and control sites may bias intervention estimates. In addition, although the original interventions have shown promising results in previous studies, the impact of the interventions might be more modest in a real-life setting. This dilution of intervention effect is a common finding in effectiveness studies and implementation trials (66), however the iterative approach to the implementation in our study is meant to reduce the chance of such dilution (54, 66).

A major strength of this study is the established efficacy in the original four interventions, the co-creation with the municipality, ECECs and MCH undertaken, and the established capacity and networks built by the research team. This experience will help address challenges along the way. The municipality setting and organization of ECEC and MCH are quite similar in all parts of Norway and thus, we expect that our findings related to the implementation will be generalizable to other Norwegian municipalities. Further, focusing on culturally diverse groups both in the effectiveness and implementation part, will render more culturally diverse and sensitive resources for future approaches.

*Nutrition Now* will break new ground by (i) systematically implementing and evaluating cumulative effects of successive dietary interventions in the first 1000 days of life in a real-life context, (ii) maximising interdisciplinary collaboration and integrating an anthropological approach with quantitative methods to truly adapt the intervention to context, (iii) bridging the previously described evidence-to-practice gap through rigorous scientific effectiveness evaluation of municipal implementation, (iv) harnessing the benefits of digital, video-based, technology to optimise access to dietary guidance in the municipalities, and (v) taking advantage of existing settings across sectors in municipal care to secure universal reach.

Lifecourse research has tended to be skewed towards epidemiology rather than implementation, and commonly target single transitional phases (67). Our project addresses these scientific shortcomings by implementing and evaluating evidence-based e-learning resources across multiple transitions early in life in a real-world community setting. Few developed interventions proven successful in controlled experimental settings are used to their full potential. Our study integrates four efficacious dietary interventions comprising e-learning resources and applies them in a municipal care setting. If this approach demonstrates public health benefit without increasing care providers’ workload it may provide a valuable contribution to other fields of lifecourse research.

Norway alone has the potential to save 14.8 billion EUR annually in health costs from improvements in population diet (68). According to Norwegian public health law, municipalities and counties are responsible for providing sound public health advice on diet and nutrition (69). MCH nurses and midwives are in a key position to guide parents on diet and nutritional issues, but often lack resources and time (70). ECEC staff are also strategically important as they are responsible for care of children while their parents work or study. Our digital diet interventions (70–72) offer a low-cost complementary tool for public health work in municipalities, and if successfully adapted when scaled-up, have the potential to improve population diet and health and respond to the need for tools and improved skills in childcare and ECECs. By gaining in-depth understanding of factors critical for successful local implementation, we will be ready to advance to county and national implementation, thereby contributing significantly to evidence-based health services in all municipalities in Norway.

## Data availability statement

We plan to share anonymised data in the UiA deposit Dataverse.

## Ethics statement

The studies involving human participants were reviewed and approved by Regional Ethics Committee (REC), South-East, reksorost@medisin.uio.no, Reference number: 322480, and our Faculty Ethical Committee (FEC) and the Norwegian Data Protection Service (NSD) (id-number: FEC. NSD:847590). Written informed consent to participate in this study was provided by the participants’ legal guardian/next of kin.

## Author contributions

NØ, EH, FN, AM contributed to the conception of Nutrition Now project. NØ is the PI of the project, with AM, EH and FV as co-PIs. All authors contributed to the design of the study. SH, CH, EV, and EH contributed substantially to the development of the Nutrition Now resource, led by NØ. NO, HL, SH, CH, UV, PL, KC, CK, AM, EH, HR, and NØ contributed to the implementation trial, led by FV. NO, HL, CH, MA, EV, EH and NØ contributed to the effectiveness trial, led by AM. TE, MB and NØ contributed to the planning of socio-economic and ethnic diversity focus, led by MG and WV, respectively. AW contributed substantially to data management with EG, led by TB. Work on statistical approaches is led by AM and AW. AL contributed substantially to the cost-effectiveness part, led by EH. NØ developed the first draft of the manuscript, which was further developed by AM, FV, AW and EH. All authors contributed to the article and approved the submitted version.
