# Supplementary material for: Evaluating the effectiveness and implementation of evidence-based early-life nutrition interventions in a community setting a hybrid type 1 non-randomized trial – the Nutrition Now project protocol
Source: Front Endocrinol (Lausanne). 2023 Jan 10;13:1071489. doi: 10.3389/fendo.2022.1071489 (PMC9871808; doi:10.3389/fendo.2022.1071489)
Supplement: Supplementary file 1 [file Table_1.docx]

**Table S1** Description of aim, intervention content and results from the four original interventions included in Nutrition Now (Additional file 1)

| RCT - Project | Aim | Intervention content | Results |
| --- | --- | --- | --- |
| **The Norwegian Fit for delivery study**  (1) | To evaluate the effect of a lifestyle intervention in pregnancy on maternal and child health outcomes. | **Control group:** standard prenatal care **Intervention group:**   - Standard prenatal care + information on healthy eating and physical activity provided in pamphlets - 10 dietary recommendations targeting energy balance-related behaviors - Nutritional counseling by phone - Access to twice-weekly exercise sessions - Evening meetings (one with additional information about the trial and one with a hands-on cooking class)   No specific theory of change | Women in the intervention group of the NFFD study reported   - Higher relative consumption of water - More frequent fruit and vegetable consumption - Less frequent consumption of sugar-dense foods - Buying smaller packages of unhealthy foods - Less frequent overeating - Reading food labels more often than control women post-intervention   The Norwegian Fit for Delivery lifestyle intervention in pregnancy had no significant effect on obstetrical or neonatal outcomes but did show a modest but significant decrease in gestational weight gain (2). |
| **Early food for future health**  (3) | To evaluate the effect of an e-learning intervention (BarnE-mat) aiming to promote a healthy and sustainable diet and healthy food habits in children through encouraging beneficial parental feeding practices and enhancing parental self-efficacy regarding knowledge, skills, and confidence with respect to child feeding. | **Control group:** standard infant care  **Intervention group:**   - Standard infant care + access to the website - Received monthly e-mails with links to an age-appropriate website when their child was between 6 and 12 months. - In total, seven monthly video clips of 3-5 minutes duration, focusing on feeding-related aspects such as appropriate food types and textures, how taste-preferences evolve, responsive feeding practices, and monthly cooking-films and recipes demonstrating how to make homemade baby- and family food from easily available ingredients   Theory of change:  Elements from attachment-theory, social cognitive theory and the framework of anticipatory guidance to promote knowledge about infant nutrition and feeding behavior:   1. Attachment-theory 2) Social cognitive theory 3) Anticipatory guidance | A total of 89% of the intervention group reported viewing all/most of the videoclips and responded that they were well adopted to the child’s age.  Children in the intervention group were served vegetables/fruits more frequently and had tasted a wider variety of vegetables compared to control. Intervention children were more likely to eat family breakfast and dinner, and less likely to be playing or watching TV/tablets during meals (4).  At child age 24 months there was no evidence of sustained intervention effects, but the dietary patterns and mealtime routines at child age 24 months were reasonably consistent and in the same direction as at child age 12 months (5). |
| **Food 4 Toddlers**  (6) | To evaluate the effect of an e-health intervention which aimed to promote healthy dietary habits in toddlers by targeting parents’ awareness of their child’s food and eating environments. | **Control group:** standard toddler care  **Intervention group:**   - Usual care + Access to the food 4 toddlers webpage including modules covering an introduction and seven topics on promoting a healthy food and eating environment for the child, recipes, a discussion forum   Theory of change:  1) Strengthen positive beliefs, weaken negative beliefs, introduce new beliefs 2) Active learning through activity-based experiences – videos 3) Persuasive communication – emphasis on small changes, familiar settings 4) Modeling – actors modeling desired behaviors | The difference between groups in the change from baseline to follow-up 1 was 0.46 vegetable items per day in favor of the intervention group. The intervention effect was attenuated and no longer significant 6 months post intervention.  The process evaluation revealed that 87% of the participants in the intervention group visited the website. Most parents found the website appropriate to the child’s age as well as self-explanatory. The recipes were valued as the most appreciated element included in the website (7). |
| **Children’s food courage 2.0**  (8) | To evaluate the effect of two different interventions arms among one-year old children in ECEC to reduce food neophobia and promote healthy diets | **Control group:** Continued usual meal practice in ECEC.  **Intervention group 1:**   - Serve three alternating warm lunch dishes with a variety of vegetables, three days a week during the intervention period of three months. In total, children were exposed to three different menus, and had 1 week off, before they started the new menu for three weeks - The recipes were available on a webpage   **Intervention group 2:**   - Same intervention as group 1 + - ECEC staff implemented pedagogical tools: Weekly sensory lessons (Sapere method). - Advice on meal practice and feeding practices during mealtime. Meal practice and feeding practice recommendations were presented in short information videos on the study web page which was only accessible for this intervention group - Staff were encouraged to show the intervention videos to the children's parents.   Theory of change:  Socio-cognitive theory and the socio ecological model | Higher intake of the three intervention vegetables in group 2 (diet + Sapere/pedagogical tools). Weak suggestion that the total vegetable intake increased in group 1 (diet). No detectable effects on food neophobia (9).  Five main themes were identified from qualitative interviews with ECEC staff: 1) One-year-olds love food and renewal of the menus was inspiring; 2) One-year-olds are surprisingly willing to try and accept novel foods; 3) Novel food at meals stimulate social interaction; and 4) The Sapere method is a fun and explorative activity for 1-year-olds. These four themes were the features perceived as the effective elements of the intervention by the kindergarten teachers. The fifth main theme was: 5) Sustained impact on kindergarten teachers' practices and beliefs. The kindergarten teachers found the intervention easy to implement, and they were surprised by the foods 1-year-olds like and how the intervention increased their food acceptance. (10) |

1. Sagedal LR, Øverby NC, Lohne-Seiler H, Bere E, Torstveit MK, Henriksen T, et al. Study protocol: fit for delivery - can a lifestyle intervention in pregnancy result in measurable health benefits for mothers and newborns? A randomized controlled trial. BMC Public Health. 2013;13:132.

2. Sagedal L, Øverby N, Bere E, Torstveit M, Lohne-Seiler H, Småstuen M, et al. Lifestyle intervention to limit gestational weight gain: the Norwegian Fit for Delivery randomised controlled trial. BJOG: An International Journal of Obstetrics & Gynaecology. 2017;124(1):97-109.

3. Helle C, Hillesund ER, Omholt ML, Øverby NC. Early food for future health: a randomized controlled trial evaluating the effect of an eHealth intervention aiming to promote healthy food habits from early childhood. BMC Public Health. 2017;17(1).

4. Helle C, Hillesund ER, Wills AK, Øverby NC. Evaluation of an eHealth intervention aiming to promote healthy food habits from infancy -the Norwegian randomized controlled trial Early Food for Future Health. International Journal of Behavioral Nutrition and Physical Activity. 2019;16(1).

5. Helle C, Hillesund ER, Wills AK, Øverby NC. Examining the effects of an eHealth intervention from infant age 6 to 12 months on child eating behaviors and maternal feeding practices one year after cessation: The Norwegian randomized controlled trial Early Food for Future Health. PLOS ONE. 2019;14(8):e0220437.

6. Røed M, Hillesund ER, Vik FN, Van Lippevelde W, Øverby NC. The Food4toddlers study - study protocol for a web-based intervention to promote healthy diets for toddlers: a randomized controlled trial. BMC Public Health. 2019;19(1).

7. Røed M, Vik FN, Hillesund ER, Van Lippevelde W, Medin AC, Øverby NC. Process Evaluation of an eHealth Intervention (Food4toddlers) to Improve Toddlers' Diet: Randomized Controlled Trial. JMIR Human Factors. 2020;7(3):e18171.

8. Blomkvist EAM, Helland SH, Hillesund ER, Øverby NC. A cluster randomized web-based intervention trial to reduce food neophobia and promote healthy diets among one-year-old children in kindergarten: study protocol. BMC Pediatrics. 2018;18(1).

9. Blomkvist EAM, Wills AK, Helland SH, Hillesund ER, Øverby NC. Effectiveness of a kindergarten-based intervention to increase vegetable intake and reduce food neophobia amongst 1-year-old children: a cluster randomised controlled trial. Food & Nutrition Research. 2021;65.

10. Helland SH, Øverby NC, Myrvoll Blomkvist EA, Hillesund ER, Strömmer S, Barker M, et al. Wow! They really like celeriac! Kindergarten teachers' experiences of an intervention to increase 1-year-olds' acceptance of vegetables. Appetite. 2021;166:105581.
