# Supplementary material for: Evaluating the effectiveness and implementation of evidence-based early-life nutrition interventions in a community setting a hybrid type 1 non-randomized trial – the Nutrition Now project protocol
Source: Front Endocrinol (Lausanne). 2023 Jan 10;13:1071489. doi: 10.3389/fendo.2022.1071489 (PMC9871808; doi:10.3389/fendo.2022.1071489)
Supplement: Supplementary file 2 [file Table_2.docx]

**Table S2** Core components in the Nutrition Now resource (Additional file 3)

| **Nutrition Now Core Component** | **Age-appropriate reinforcement of core component information** | | | | **ECEC** |
| --- | --- | --- | --- | --- | --- |
|  | **Pregnancy** | **Newborn** | **Infants**  5-12 months | **Toddlers**  12-24 months | **ECEC**  1-3 years |
| **A healthy diet is important early in life**  This is an overarching message provided in several components of the intervention, however also addressed by itself, explaining the importance of food, diet quality and variety from pregnancy throughout the first two years. | Addressed in videos delivered at two timepoints during pregnancy, approx. gestational week 8 and 12. |  | Addressed in all videos. | Addressed in the majority of the messages*.* | Addressed in most messages to staff*.* |
| **Breastfeeding is best for the baby and for the benefit of the mother**  Breastmilk is the optimal nutrition in the first 6 months of life. It provides energy, nutrients and a multitude of bioactive substances that is important for healthy development. Further, breastmilk transfers flavours from maternal diet, facilitating acceptance of a varied diet. Lastly, breastfeeding represents a unique setting facilitating bonding between mother and child. | Addressed in a video to be shown in third trimester around pregnancy week 28. | Addressed in a video to be shown shortly after birth to reinforce the message about the benefits of breastfeeding. | Addressed in the first video with focus on the value of sustained breastfeeding along with the introduction of solid food. |  |  |
| **Parents play a key role in food provision and in shaping healthy eating habits for the child**  To develop healthy eating habits, the child needs regular and repeated exposure to a varied and healthy diet in a predictable and safe eating environment shaped by their caregivers. | Addressed in a video around gestational week 16. |  | Addressed in most of the videos. | Addressed in most of the messages*.* | ECEC staff’s role is addressed in several videos and messages. |
| **Responsive feeding supports the child’s internal regulation of energy intake**  Infants can self-regulate their energy intake by responding to internal signals of hunger and satiety. When caregivers respond to infant feeding cues in a prompt and developmentally appropriate manner, the infant’s regulatory capacity and feeding autonomy is enhanced. In contrast, feeding practices that override child hunger and satiety cues are associated with less beneficial weight development.  Non-responsive feeding practices comprise using food as reward, pressure to eat, control of food intake and using food to regulate child feelings. |  | Addressed in two videos about breastfeeding and bottle feeding, respectively, to be shown shortly after birth. | Addressed in most of the videos. | Addressed in several messages. | Addressed in messages and one video to staff. |
| **The shared meal is a cornerstone in the child’s eating environment**  The family meal is an important arena for the child’s eating development and eating habits. Food culture, dietary behaviours and taste preferences are transferred in the meal setting among others through role-modelling by parents and siblings.  In addition, a predictable mealtime structure along with a positive atmosphere around meals, make the child feel safe and secure. This promotes enjoyment of food as well as learning and social development. | Addressed in a video around gestational week 16, focusing on the value of establishing regular meals before the baby is born. |  | Addressed in several videos. | Addressed in several messages. | ECEC staff are given advice on how to use the meal setting as a time to learn to like new foods and for food enjoyment. Staff are given messages related to creating a positive atmosphere and encouraging children to use their senses and participate actively. |
| **Knowledge and experience with food preparation and cooking is important for healthy eating in everyday family-life**  Lack of knowledge and skills regarding food preparation and cooking may be barriers in food parenting. Providing ‘easy-to-grasp’ information and ‘easy-to-make’ recipes are useful in building parental competence and self-efficacy. | Addressed in a video around gestational week 16, including recipes of healthy meals and short films demonstrating easy and time-efficient cooking. | Recipes and/or cooking videos are included, focusing on ‘easy-to-make’ and ‘easy-to-eat’ meals in the early postpartum period. | Recipes and/or cooking videos are included in all monthly messages. | Recipes and/or cooking videos are included in all messages. | Recipes for healthy lunches, including repeated exposures of certain vegetables, are forwarded monthly. Instruction videos and messages show how the ECEC staff can carry out easy and time-efficient cooking.  Advice on following the national nutritional guidelines is provided. |
| **Sensory play with vegetables**  Sensory play in educational / playful sessions, detached from the usual meal context, gives children opportunity to explore foods and vegetables in a systematic way in combination with wonder and inquisitiveness. Experiencing food with five senses - smell, sight, touch, hearing and taste - contributes to sensory awareness, language and familiarity with new foods (the Sapere method). Sensory sessions in early age can increase enjoyment of vegetables and thereby affect child's eating habits. |  |  |  |  | A template, videos and messages for facilitating weekly Sapere sessions in ECEC are included. |
| **ECEC collaboration with parents** Profiling lunch dishes and staff feeding practices provides parental insight that makes it easier to ask questions and exchange observations and assessments related to the child's eating development, health and well-being. |  |  |  |  | Addressed in one video and several messages to staff. |

ECEC: Early Childhood Education and Care
